# Hemicrania continua with contralateral cranial autonomic features: a case report

**DOI:** 10.1186/s10194-015-0508-6

**Published:** 2015-03-11

**Authors:** Sanjay Prakash, Chaturbhuj Rathore, Prayag Makwana

**Affiliations:** Department of Neurology, Smt B. K. Shah Medical institute and research Centre Medical College, Piperia, Waghodia, Vadodara, Gujarat 391760 India

**Keywords:** Hemicrania continua, Cluster headache, Paroxysmal hemicrania, Trigeminal autonomic cephalagias, Indomethacin

## Abstract

**Background:**

Hemicrania continua is characterized by continuous strictly unilateral head pain with episodic exacerbations. Episodic exacerbations are associated with ipsilateral cranial autonomic features.

**Case description:**

We report a 24-year female with a 2-year history of continuous right-sided headache with superimposed exacerbations. Episodic exacerbations were associated with marked agitation and contralateral cranial autonomic features. The patient showed a complete response to indomethacin within 8 hours.

**Discussion:**

The dichotomy of pain and autonomic features is in accordance with the concept about the possibility of two separate pathways for pain and autonomic features in trigeminal autonomic cephalalgias.

## Background

Hemicrania continua (HC) is characterized by continuous strictly unilateral headache with superimposed episodic exacerbations. Patients show dramatic and complete response to therapeutic doses of indomethacin [1]. The frequency and duration of exacerbations are highly variables. Exacerbations are usually associated with autonomic disturbances on the painful side of the head [2]. Herein we describe a case of HC who had autonomic features only on the contralateral side. We also speculate pathophysiology for such association.

## Case presentation

A 24-year-old female presented with a 2-year history of continuous right-sided headache with superimposed exacerbations. The continuous pain was dull, mild - moderate severity, and maximal in the retro-orbital and supraorbital areas. The exacerbations, described as excruciating pulsatile pain, occurred every alternate day to 4 attacks in a day and lasted for 30 minutes to 4 hours. Exacerbations were graded as 9-10 on visual analogue scale (VAS). Exacerbations were associated with marked restlessness or agitation in the form of hitting head against wall, pacing activity and crying. The patient reported cranial autonomic feature during exacerbations, but it was only on the contralateral to the pain side. Cranial autonomic features were conjunctival injection, tearing and eyelid edema. The autonomic features were never on the side of the pain. Nausea and phonophobia were noted occasionally during exacerbations. Nocturnal attacks were also reported. There were no precipitating or aggravating factors for the exacerbations. Family history of headache was negative. Past treatments include drugs: amitriptylin, propranolol, topiramate, flunarizine, sodium valproate, Naproxen, diclofenac, paracetamol, and ibuprofen. None of them provided any significant improvement. Physical examinations and investigations (including MRI brain) were normal.

The patient was admitted and was put under observation for her claim of contralateral cranial autonomic features. There were two exacerbations in the next 36 hours. We confirmed the patient’s claim and witnessed conjunctival injection, tearing and eyelid edema in both exacerbations. A diagnosis of atypical HC was made and indomethacin was started at the dose of 25 mg three times daily and the patient showed complete improvement within 8 hours. The patient had never felt such improvement by any drug since the beginning of her clinical presentation.

Tapering was done after three months, but it was not successful. Tapering or skipping of the drug led to recurrence of symptoms within 6- 12 hours. Recurrences were associated with contralateral cranial autonomic features. No side effect was reported by patient in the next 6 months follow up.

## Discussion

This patient fulfilled the ICHD-3β criteria for HC [1]. However, the unusual feature in the patient was the presence of cranial autonomic feature on the contralateral side. To the best of our literature search, no such patient of HC with contralateral autonomic features has been described in the literature. There are few case reports of bilateral HC in the literature where patients had autonomic features on both sides with exacerbations [3]. HC was also reported with other TACs on the contralateral side with cranial autonomic feature [4]. Our patient did not have pain on the left side and cranial autonomic features were noted only during the exacerbation period. It suggests that the patient had only one type of headache.

As far as other TACs are considered, there is just one case report of cluster headache (CH) in which Horner’s syndrome was noted on the contralateral side of pain [5]. In another patient with side-shifting CH, cranial autonomic features were noted on the original side of headaches (i.e. opposite to the side of pain of the recent attacks) [6].

HC and other TACs are known for the presence of ipsilateral cranial autonomic features. It has been proposed that cranial autonomic features in the TACs are because of disinhibition of trigeminal autonomic reflex (TAR) by the hypothalamus [7]. Hypothalmic abnormalities have been noted in all kind of TACs. Hypothalamic activation has been reported as ipsilateral to the headache in CH and contralateral to headache in PH and HC [7,8]. There is suggestion that ipsilateral activation of the hypothalamus during cluster headache attacks stimulate ipsilateral but simultaneously suppress contralateral TAR [9]. The same mechanism could be speculated for other TACs. The autonomic symptoms are mainly related to increased parasympathetic outflow which is mediated by sphenopalatine and otic ganglia. A recent study by Schyt et al [10] demonstrated that low frequency SPG stimulation induce cluster-like attacks with autonomic features, while high frequency SPG stimulation suppress it. This observations indicate that stimulation of SPG also vary with stimulation parameters. In other observation Akerman et al [11] have shown that antinociceptive effects of oxygen inhalation is mediated through parasympathetic fibers. Collectively, these data suggest that cranial parasympathetic fibers facilitate both pain and cranial autonomic features.

However, the interrelation of cranial autonomic features with pain attacks are highly variables. Cranial autonomic features are not universal in all TACs (except SUNCT/SUNA) and do not always occur. About 7% patients with CH might not have cranial autonomic features [8]. As far as HC is concerned, up to one third of patients with HC might not have autonomic features during exacerbations [2]. Interestingly, a few patients with CH and PH have been reported with episodic characteristics autonomic features but no headache [7]. Episodic cranial autonomic symptoms without headache have been reported in CH patients even after trigeminal nerve root section [12]. These observations suggest that the headaches and autonomic features are not inextricably linked and the autonomic features might not be entirely because of TARs [8]. It is further suggested that the nerve pathways mediating these two features are likely to be at least partly separate [8].

Cranial autonomic feature is classically described ipsilateral to headache. However, many patients may have bilateral CAS (but predominantly on ipsilateral side) [13]. A few studies suggested that trigeminal autonomic reflex may include some fibers from contralateral side, due to the crossover in the brainstem [14]. This could explain the bilateral CAS in CH and other TACs.

A few assumptions could be made for the dichotomy of pain and autonomic features in our patient. Although cranial autonomic features were noted on the opposite side, it was time locked with exacerbations phase, suggesting a common generator for both pain and autonomic features. However, the pain on one side and autonomic features on the contralateral side suggest that both pain and autonomic features have two separate pathways. This is in accordance with the assumption of a few authors who believe that there could be two separate pathways for pain and autonomic features [8]. Episodic cranial autonomic symptoms without a headache in CH patients even after trigeminal nerve root section also suggest the same assumption [12].

Cranial autonomic features on the contralateral side may be because of the stimulation of crossover fibers of trigeminal autonomic reflex in the brainstem. As noted earlier that ipsilateral activation of the hypothalamus during cluster headache attacks stimulate ipsilateral but simultaneously suppress contralateral trigeminal autonomic reflex [9]. As hypothalamic abnormalities are contralateral to the side of pain in HC, it could be speculated that in HC, hypothalamus stimulate contralateral and suppress the ipsilateral trigeminal autonomic reflex. It means that hypothalamus has potential to stimulate and suppress to any side of TAR. In this case, hypothalamus has stimulated ipsilateral TAR and simultaneously suppressed contralateral TAR.

## Conclusion

Hemicrania continua and other TACs may have contralateral autonomic features.

Dichotomy of pain and autonomic features suggest two separate pathways for these features.

## Consent

Written informed consent was taken from the patient for the publication of this case report.

## References

[1] Headache Classification Committee of the International Headache Society (2013). The international classification of headache disorders, 3rd edition. Cephalalgia.

[2] Prakash S, Golwala P (2012). A proposal for revision of hemicrania continua diagnostic criteria based on critical analysis of 62 patients. Cephalalgia.

[3] Southerland AM, Login (2011). IS. Rigorously defined hemicranias continua presenting bilaterally. Cephalalgia.

[4] Totzeck A, Diener HC, Gaul C (2014). Concomitant occurrence of different trigeminal autonomic cephalalgias: A case series and review of the literature. Cephalalgia.

[5] Sjaastad O, Antonaci F, Fragoso YD (1988). Cluster headache: further observations on the dissociation of pain and autonomic findings. Cephalalgia.

[6] Drummond PD (1990). Dissociation between pain and autonomic disturbances in cluster headache. Headache.

[7] Prakash S, Hansen JM, Martelletti P, Timothy J, Steiner TJ (2011). Mechanisms of cluster headache and other trigeminal autonomic cephalalgias. Handbook of headache: practical management.

[8] Leone M, Bussone G (2009). Pathophysiology of trigeminal autonomic cephalalgias. Lancet Neurol.

[9] Young WB, Rozen TD (1999). Bilateral cluster headache: case report and a theory of (failed) contralateral suppression. Cephalalgia.

[10] Schytz HW, Barløse M, Guo S, Selb J, Caparso A, Jensen R, Ashina M (2013). Experimental activation of the sphenopalatine ganglion provokes cluster-like attacks in attacks in humans. Cephalalgia.

[11] Akerman S, Holland PR, Lasalandra MP, Goadsby PJ (2009). Oxygen inhibits neuronal activation in the trigeminocervical complex after stimulation of trigeminal autonomic reflex, but not during direct dural activation of trigeminal afferents. Headache.

[12] Lin H, Dodick DW (2005). Tearing without pain after trigeminal root section for cluster headache. Neurology.

[13] Baron M, Gili P, Sanchez-del-Rio M, Barriga FJ, Yangüela J, Vela L, Sánchez C, Dobato JL, Pardo FJ, Pareja JA (2007). Objective assessment of bilateral conjunctival injection during cluster headache attacks. Neurology.

[14] Lambert GA, Bogduk N, Goadsby PJ, Duckworth JW, Lance JW (1984). Decreased carotid arterial resistance in cats in response to trigeminal stimulation. J Neurosurg.

